# Prognostic factors for the outcome of needle aspiration of calcific deposits for rotator cuff calcific tendinitis

**DOI:** 10.1007/s00330-020-06669-0

**Published:** 2020-03-05

**Authors:** Bart W. Oudelaar, Rianne Huis In ‘t Veld, Relinde Schepers-Bok, Edwin M. Ooms, Rob G. H. H. Nelissen, Anne J. H. Vochteloo

**Affiliations:** 1 Centre for Orthopaedic Surgery OCON, Geerdinksweg 141, P.O. Box 546, 7550 AM Hengelo, The Netherlands; 2 Department of Radiology, Ziekenhuisgroep Twente, Hengelo, The Netherlands; 3 Department of Orthopaedics, Leiden University Medical Center (LUMC), Leiden, The Netherlands

**Keywords:** Rotator cuff, Tendinopathy, Treatment, Prognostic factors, Prospective study

## Abstract

**Objective:**

To identify prognostic factors for the effectiveness of needle aspiration of calcific deposits (NACD) for rotator cuff calcific tendinitis (RCCT)

**Methods:**

One hundred forty-nine patients with symptomatic RCCT were included in a prospective cohort study. Pain (VAS), shoulder function (SST and DASH), and quality of life (EQ-5D) were assessed at baseline and at 3, 6, and 12 months post-NACD. Univariate analyses (independent *t* tests or Mann-Whitney *U* tests depending on the distribution of data) were performed to build a multivariable linear regression model. Stepwise regression analysis through backward elimination was performed to evaluate the effect of predefined prognostic factors on the outcome.

**Results:**

Patients who underwent multiple NACD procedures had less reduction of pain (*p* < 0.01). Furthermore, a larger reduction in VAS pain scores at 3 months post-NACD was associated with a larger reduction in VAS pain scores at 12 months (*p* < 0.01). More improvement of SST and DASH scores at 3 months was associated with better SST, DASH, and EQ-5D scores at 12 months (*p* < 0.01). Smaller-size calcific deposits were associated with less improvement of DASH (*p* = 0.03) and EQ-5D scores (*p* = 0.01). A longer duration of symptoms prior to NACD was associated with less improvement of EQ-5D scores (*p* = 0.01).

**Conclusions:**

A good initial response after NACD is associated with better outcomes at 12 months. Patients with a longer duration of symptoms prior to NACD and patients who require multiple procedures showed inferior outcomes in terms of pain reduction and improvement of quality of life. Smaller-size calcific deposits are associated with a less favorable outcome of shoulder function and quality of life scores and might therefore be less susceptible for NACD.

**Key Points:**

*• A good initial response to NACD is associated with a better outcome in the longer term.*

*• A longer duration of symptoms and the need for multiple NACD procedures are associated with inferior outcomes.*

*• Smaller-size calcific deposits seem less susceptible for NACD.*

**Electronic supplementary material:**

The online version of this article (10.1007/s00330-020-06669-0) contains supplementary material, which is available to authorized users.

## Introduction

Rotator cuff calcific tendinitis (RCCT) is a frequently diagnosed shoulder disease, which is found in up to 54% of patients with shoulder complaints [1]. It is suggested that RCCT is a self-limiting disease that can be treated with conservative measures (rest, non-steroidal anti-inflammatory drugs (NSAIDs), or physiotherapy) [2–4]. However, a substantial number of patients has persisting symptoms requiring other therapies such as subacromial injections with corticosteroids, needle aspiration of calcific deposits (NACD), or extracorporeal shockwave therapy (ESWT) [5]. A recently published meta-analysis comparing these therapies concluded that NACD is the most effective treatment considering relevant clinical outcomes within a follow-up term of 2 years [6]. However, up to 42% of patients experience persisting or recurrent shoulder complaints after NACD [7, 8]. Female gender, smoking, and type I calcifications according to the classification by Gärtner and Heyer are considered predictors for persisting shoulder complaints [7, 9, 10]. However, the majority of studies investigating prognostic factors for the effectiveness of NACD are retrospective and, to our knowledge, no prospective studies investigating prognostic factors have been conducted. As more evidence is needed on this highly prevalent shoulder disease, we conducted a prospective cohort study to further identify prognostic factors for the effectiveness of NACD.

## Materials and methods

### Study design and study population

A prospective cohort study was conducted at the departments of orthopedic surgery and radiology. All consecutive patients referred to the department of radiology between October 2014 and August 2016 for the treatment of symptomatic RCCT were included. Inclusion criteria were clinical signs of calcific tendinitis defined as pain in the deltoid region worsening by elevation of the arm above the shoulder level and/or at night and the presence of calcific deposits on radiographs and/or ultrasound examination. Exclusion criteria were age < 18 years, being unable to read and write the Dutch language, previous NACD treatment or surgery of the affected shoulder, and the presence of other causes for shoulder complaints (e.g., full thickness tear of the rotator cuff, frozen shoulder). Only the treatment of the first shoulder was analyzed in patients who underwent bilateral treatment.

Since it was a prospective cohort study evaluating current treatment and follow-up standards of our department, the study was declared free from subject to the medical research involving human subjects act and the IRB approved the local study execution.

### Data collection and outcome measures

After the participating patients signed informed consent forms, baseline data, including demographics and data related to the medical history and previous treatments, were collected.

Prior to the NACD procedure, baseline scores were completed by all patients: visual analogue scale for pain (VAS 0–100 mm where 0 mm represents “no pain” and 100 mm “the worst pain ever possible”), the Simple Shoulder Test (SST; 0 (worst score) to 12 (best score)), the Disabilities of the Arm, Shoulder and Hand questionnaire (DASH; 0 (no disability) to 100 (most severe disability)), and the EQ-5D (0 (worst score) to 1 (best score)).

Every 2 weeks during the first 10 weeks following the NACD procedure, patients reported a VAS pain score, whether they had physiotherapy and whether they had used analgesic medication (type and dose).

At 3 months, 6 months, and 1 year after the NACD procedure, patients reported the VAS pain score, SST, DASH, and EQ-5D questionnaires. Furthermore, the number of NACD procedures patients underwent was noted. The indication to perform a repeated NACD procedure was persisting clinical symptoms of calcific tendinitis with calcific deposits on ultrasound examination in the absence of another cause for shoulder complaints (e.g., frozen shoulder, subacromial bursitis). A minimum period of 6 weeks between the first and second NACD procedure or the second and third NACD procedure was applied.

The primary endpoints were as follows: reduction of pain at 12 months post-NACD (∆VAS baseline-12 months); improvement of shoulder function at 12 months post-NACD (∆SST baseline-12 months and ∆DASH baseline-12 months); improvement of quality of life (QoL) at 12 months post-NACD (∆EQ-5D baseline-12 months). Minimal clinically important differences (MCID; ≥ 14 mm for VAS [11], ≥ 2.0 for SST [12], ≥ 10.83 for DASH [13], and ≥ 0.07 for EQ-5D [14]) were used to determine whether the reduction of pain and improvement of shoulder function and QoL were clinically relevant.

### Radiographic assessment and technical procedure

All patients had both radiographic and ultrasound assessment of the affected shoulder prior to the NACD procedure.

Standard anteroposterior and axial radiographs were used to examine the size and number of calcific deposits and the affected tendon(s). Calcific deposits were categorized into three different types using the classification described by Gärtner and Heyer [9]: type I calcifications are dense with well-defined borders; type II calcifications are either dense with indistinct borders or transparent with well-defined borders; and type III calcifications are transparent with indistinct borders. The location of the calcific deposits was determined based on the method used by Ogon et al [3] in which negative values represent a medial calcification border.

Ultrasound was performed using a standardized protocol by experienced musculoskeletal radiologists. The size, the number of calcific deposits, and the affected tendon(s) were recorded. Furthermore, the presence of subacromial bursitis, partial rotator cuff tears, and subacromial impingement was assessed prior to the NACD procedure.

During the NACD procedure, the consistency of the calcific deposits was assessed as either soft (toothpaste like) or hard. Furthermore, the possibility to aspirate the calcific deposit was reported.

Ultrasound-guided NACD was performed using a single-needle technique with 20- or 21-gauge needle. After maneuvering the needle into the calcific deposit, the deposits were infiltrated with lidocaine 1%, fragmented in case of a hard calcific deposit, and removed by aspiration. After completing the aspiration, the subacromial bursa was infiltrated with 4 ml bupivacaine (2.5 mg/ml; Aurobindo Pharma B.V) and 1 ml kenacort (40 mg/ml; Bristol-Myers Squibb B.V.). No specific rehabilitation program was prescribed.

### Statistical analysis

Univariate analyses (independent *t* tests in case of normally distributed data and the Mann-Whitney *U* tests in case the data was not normally distributed) were performed to build a multivariable linear regression model. Factors that approached a significant correlation (*p* < 0.10) were included for multivariable linear regression analysis.

Stepwise regression analysis through backward elimination was performed to determine factors associated with better reduction of pain, better improvement of shoulder function, and better improvement of QoL. All the previously selected factors were entered into the equation after which the factor that contributed the least (i.e., highest *p* value) was deleted. This process was continued until all factors in the equation had a significance level of *p* < 0.05.

All statistical analyses were performed using the statistical package SPSS version 20.0 (IBM).

## Results

**Fig. 1:**
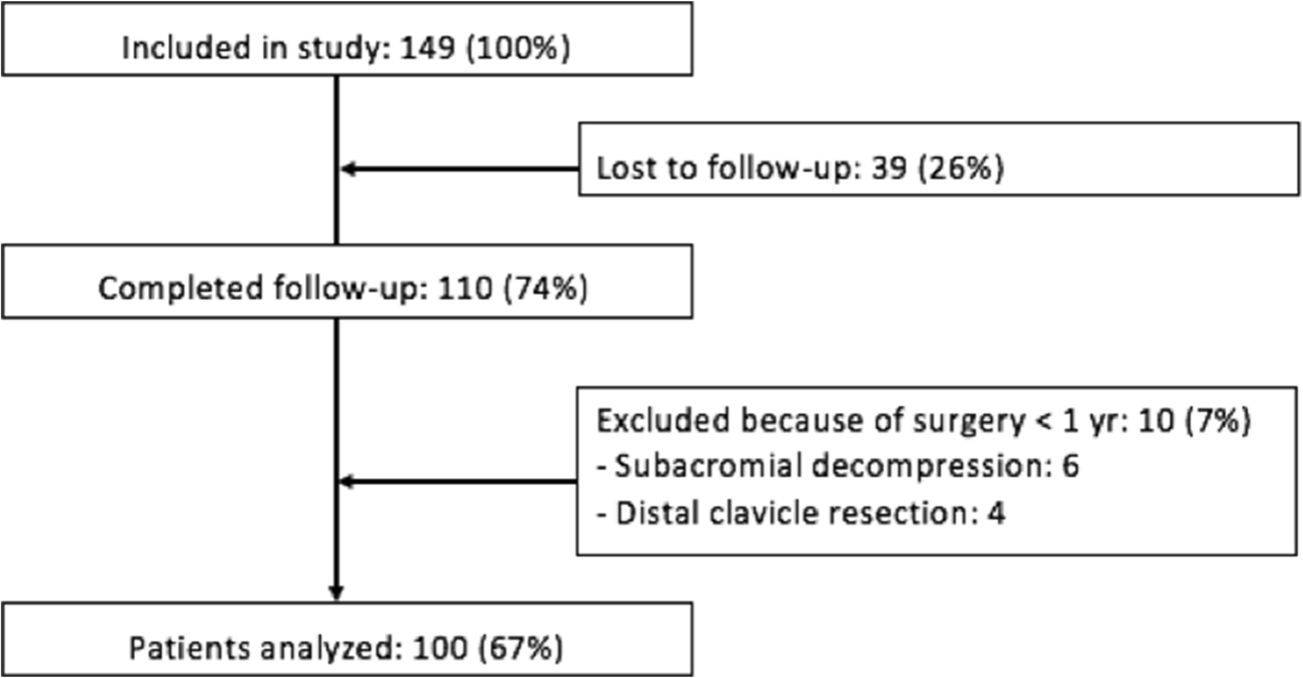
Flowchart of patient inclusion and drop-out

One hundred forty-nine consecutive patients were included of which 39 patients were lost to follow-up as these patients did not complete the 12-month follow-up questionnaires (Fig. 1). The baseline characteristics of the patients included in this study are presented in Table 1. The average duration of symptoms prior to NACD was 29 months and over one fifth of patients reported absenteeism from work due to shoulder complaints. The majority of patients underwent other therapies prior to the NACD procedure: over 75% of patients had physiotherapy and over 50% of patients already had subacromial injection(s) with corticosteroids.

**Table 1:** Baseline characteristics

Demographic details	
Male (%)	49 (45%)
Female (%)	61 (55%)
Age (SD; range)	54.9 (8.2; 36–77)
Medical history	
Duration of symptoms in months (SD; range)	29 (33; 1–120)
Bilateral occurrence (%)	25 (22.7)
Dominant arm affected (%)	65 (59.1)
Heavy physical work (%)	50 (46.3)
Absenteeism from work due to shoulder complains (%)	23 (22.5)
Average amount of days of absenteeism from work (SD; range)	78 (99; 2–360)
Smoking (%)	21 (21)
Diabetes (%)	10 (10)
Previous treatment	
Analgesics (%)	69 (62.7)
Physiotherapy (%)	84 (76.4)
Shockwave (%)	14 (12.7)
Subacromial injection (%)	59 (53.6)
Ultrasound findings	
Affected tendon(s)	
Supraspinatus (%)	103 (93.6)
Infraspinatus (%)	2 (1.8)
Subscapularis (%)	17 (15.5)
Subacromial bursitis (%)	19 (18.4)
Partial thickness rotator cuff tear (%)	27 (25.7)
Subacromial impingement (%)	8 (13.3)
Radiographic findings	
Size in mm (SD; range)	16.5 (9.4; 2.0–44.9)
Number of calcific deposits	
One (%)	61 (59.8)
Multiple (%)	41 (40.2)
Gartner and Heyer classification	
Type I (%)	60 (58.2)
Type II (%)	29 (28.2)
Type III (%)	14 (13.6)
Location according to Ogon et al in mm (SD; range)	− 6.4 (13.1; − 36.1–26.4)
Features of AC osteoarthrosis (%)	48 (44.4)
Post-NACD	
Analgesics during first 6 weeks post-NACD (%)	57 (56.4)
Physiotherapy during first 6 weeks post-NACD (%)	33 (33.0)
Average amount of physiotherapy visits (SD; range)	3.5 (2.5; 1–11)

*SD*: standard deviation

Except for age (55 years in the analyzed group vs. 51 years in the drop-out group; *p* = 0.016), none of the other demographic or baseline values differed significantly between these groups.

During the course of the study, 10 patients failed NACD treatment and had arthroscopic surgery due to persisting symptoms (Fig. 1). In the patients requiring surgery, bilateral occurrence of RCCT was more common (5/10 vs. 20/100, Fisher’s exact test *p* < 0.046) and these 10 patients had less pain relief at 3 months post-NACD compared to the non-failed group ((∆VASbaseline-VAS3months 9 mm vs. 33 mm, independent *t* test *p* < 0.043). None of the other prognostic factors studied in this study differed significantly between patients that required surgery and those who did not.

During the NACD procedure, successful aspiration of calcium was achieved in 51% (50/98 patients) of patients.

### Improvement of pain, function, and QoL at 3, 6, and 12 months

At 12 months follow-up, 83% (91/110) of patients had lower pain scores compared to their preintervention baseline score and 70% (77/110) of patient reached MCID. The average decrease of VAS scores was 31 mm (SD 24.3).

With regard to function, respectively 74% (81/110) and 78% (86/110) of patients showed improved SST and DASH scores (average improvement SST 3.6 (SD 3.5); average improvement DASH 17.5 (SD 15.9)) and respectively 65% (72/110) and 64% (70/110) of patients reached MCID. QoL improved in 56% (62/110) of the patients and remained the same in 24% (26/110); 46% (51/110) of patients showed a clinical important improvement. The average improvement in EQ-5D was 0.12 (SD 0.26).

**Fig. 2:**
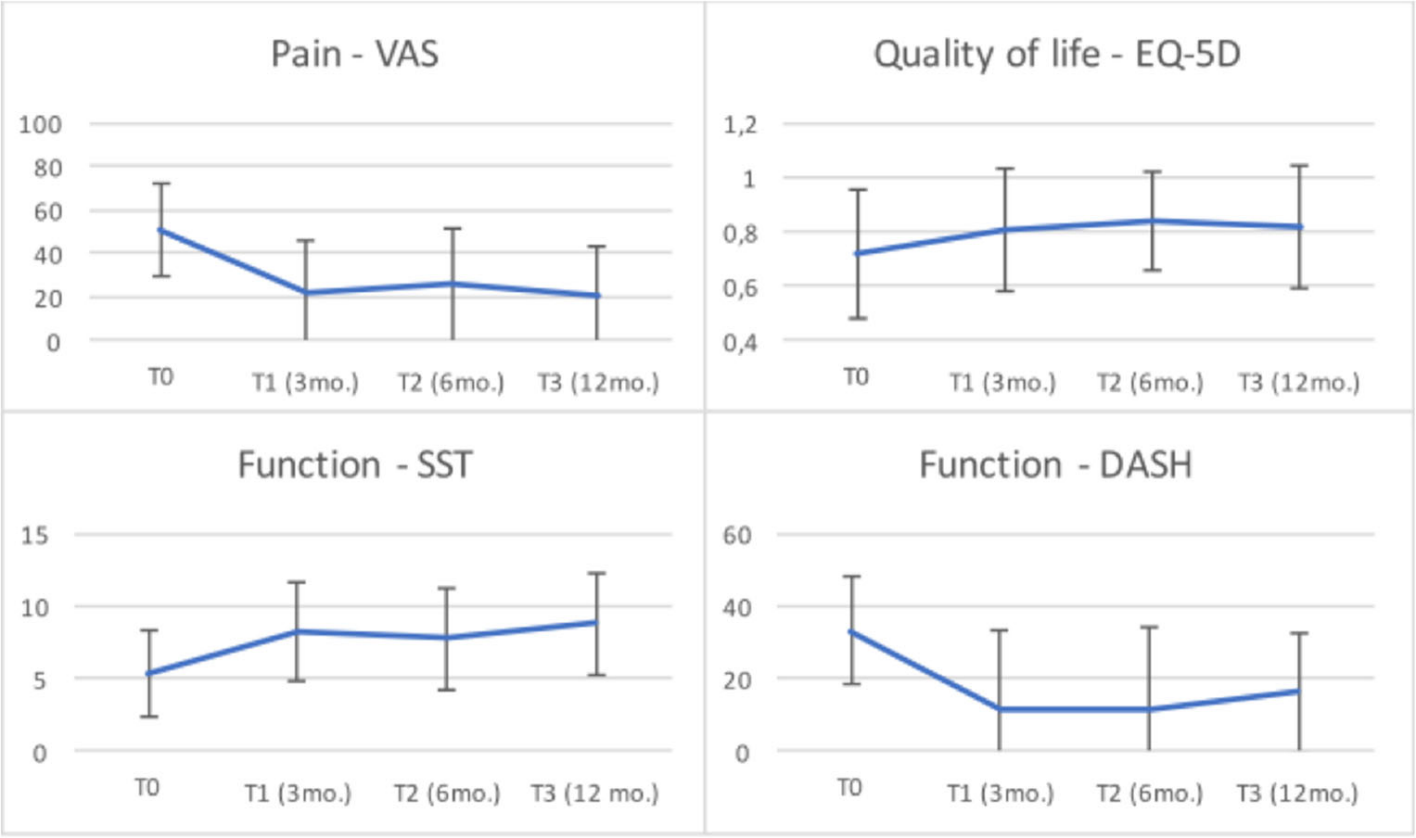
Course of pain (VAS), shoulder function (SST and DASH), and QoL (EQ-5D) during the course of this study

The largest decrease of pain, improvement of function, and QoL was seen at 3 months following the NACD procedure after which the level of pain, shoulder function, and QoL scores remained within the MCID interval (Fig. 2).

### Factors associated with decrease of pain, improvement of shoulder function, and improvement of QoL

A summary of the results of the univariate analyses for factors related to decrease of pain, improvement of shoulder function, and QoL is presented in Table 2. A complete overview of the results of the univariate analyses can be found in supplementary file I.

**Table 2:** Summary of univariate analyses for reduction of pain (VAS), improvement of shoulder function (SST and DASH), and improvement of QoL (EQ-5D)

	Factor		Value	*p* value
Pain—VAS	Multiple NACD procedures	Mean difference (95% CI)	15.5 mm (5.1 to 25.9 mm)	0.004
	ΔVAS at 3 months	Pearson correlation coefficient (95% CI)	0.500 (0.327 to 0.674)	< 0.001
	ΔDASH at 3 months	Pearson correlation coefficient (95% CI)	0.267 (0.074 to 0.461)	0.007
Function—SST	Size of calcific deposit	Pearson correlation coefficient (95% CI)	0.232 (0.028 to 0.425)	0.029
	Multiple NACD procedures	Mean difference (95% CI)	1.8 (0.3 to 3.3)	0.023
	ΔSST at 3 months	Pearson correlation coefficient (95% CI)	0.362 (0.176 to 0.549)	< 0.001
	ΔDASH at 3 months	Pearson correlation coefficient (95% CI)	0.282 (0.09 to 0.474)	0.004
Function—DASH	Absenteeism from work due to shoulder complaints	Mean difference (95% CI)	9.3 (− 1.1 to 19.7)	0.078
	Analgesics use prior to the NACD procedure	Mean difference (95% CI)	5.6 (− 0.4 to 11.6)	0.067
	Physiotherapy prior to the NACD procedure	Mean difference (95% CI)	8.6 (1.3 to 15.8)	0.021
	Subacromial impingement	Mean difference (95% CI)	11.1 (− 0.9 to 23.1)	0.068
	Size of calcific deposit	Pearson correlation coefficient (95% CI)	0.225 (0.022 to 0.413)	0.031
	Multiple NACD procedures	Mean difference (95% CI)	8.2 (1.4 to 15.1)	0.019
	ΔSST at 3 months	Pearson correlation coefficient (95% CI)	0.267 (0.074 to 0.460)	0.007
	ΔDASH at 3 months	Pearson correlation coefficient (95% CI)	0.441 (0.261 to 0.621)	< 0.001
Quality of life—EQ-5D	Duration of symptoms	Pearson correlation coefficient (95% CI)	0.246 (0.051 to 0.443)	0.026
	Size of calcific deposit	Pearson correlation coefficient (95% CI)	0.254 (0.052 to 0.452)	0.018
	Multiple NACD procedures	Mean difference (95% CI)	0.10 (− 0.01 to 0.21)	0.088
	ΔSST at 3 months	Pearson correlation coefficient (95% CI)	0.262 (0.069 to 0.456)	0.008
	ΔDASH at 3 months	Pearson correlation coefficient (95% CI)	0.185 (− 0.012 to 0.382)	0.063

*CI*: confidence interval. Mean differences were calculated using independent *t* tests or Mann-Whitney *U* tests depending on the distribution of data

Only factors with a *p* value of 0.10 or less are presented in this table. For a complete overview of all factors, see supplementary file I

With regard to reduction of pain, multiple NACD procedures were associated with more pain 12 months after the intervention. A greater reduction of pain at 3 months after NACD was associated with more pain relief at 12 months (Table 3). For the improvement of shoulder function and QoL, improved SST and DASH scores at 3 months were strongly associated with more improvement of the shoulder function and QoL at 12 months. A longer duration of symptoms prior to the NACD procedure was associated with less improvement of QoL. The size of the calcification was positively associated with DASH and QoL scores: larger size calcific deposits showed better outcome at 12 months (Table 3).

**Table 3:** Results of multivariable regression analysis for reduction of pain (VAS), improvement of shoulder function (SST and DASH), and improvement of QoL (EQ-5D)

	Factor	Comparison	Regression coefficient (95% CI)	*p* value
Pain—VAS	Number of NACD procedures	Multiple procedures vs. single procedure	− 12.34 (− 21.47 to − 3.22)	0.009
	ΔVAS at 3 months	Per 1 mm	0.409 (0.262 to 0.555)	< 0.001
Function—SST	ΔSST at 3 months	Per point	0.258 (0.134 to 0.382)	< 0.001
	ΔDASH at 3 months	Per point	0.048 (0.019 to 0.078)	0.002
Function—DASH	ΔSST at 3 months	Per point	0.692 (0.124 to 1.26)	0.018
	ΔDASH at 3 months	Per point	0.323 (0.196 to 0.449)	< 0.001
	Size of calcific deposit	Per 1 mm increase in size	0.313 (0.028 to 0.598)	0.032
Quality of life—EQ-5D	Duration of symptoms	Per months increase of symptoms	− 0.002 (− 0.003 to 0.000)	0.012
	Size of calcific deposit	Per 1 mm increase in size	0.006 (0.001 to 0.011)	0.011
	ΔSST at 3 months	Per point	0.012 (0.003 to 0.022)	0.013
	ΔDASH at 3 months	Per point	0.003 (0.001 to 0.005)	0.013

## Discussion

Our prospective study on clinical and radiographic predictors for the outcome after NACD for rotator cuff calcific tendinitis showed that the majority of patients had less pain and improvement of both shoulder function and quality of life (QoL) 1 year after NACD. A good initial response after NACD was associated with a better outcome at 12 months, whereas patients who required multiple procedures and patients with a longer duration of symptoms prior to the NACD procedure showed less favorable results. Furthermore, smaller-size calcific deposits were associated with worse shoulder function outcome and less improvement of QoL.

In our study, 70% of patients had a clinical relevant decrease of VAS pain scores. Furthermore, 64 to 65% of patients showed clinically important improvement of shoulder function, respectively measured by the SST and DASH. In 46% of the patients, QoL scores were clinically relevant improved at 1 year after NACD. The largest decrease in pain and improvement of shoulder function and QoL was seen at 3 months post-intervention after which the improvement seemed to stabilize (Fig. 2). A similar pattern of decrease in pain scores and improvement of shoulder function was found by Serafini et al [15]; the largest decrease of pain and improvement of shoulder function was also seen after 3 months after which the level of pain and shoulder function stayed relatively stable to up to 10 years of follow-up. The importance of this initial response to NACD seems to be one of the most important prognostic factors for a good outcome at 1 year. Thus, patients with less decrease of pain after NACD seem to have a higher likelihood of persisting pain at 1 year follow-up. Accordingly, patients with less improvement of the SST and DASH scores at 3 months report worse functional scores at 1 year follow-up. Thus, short-term results of NACD seem to be a good predictor for the long-term outcome which is useful in the evaluation of the treatment and in the management of patient expectations. This is a new finding compared to previous studies.

Prognostic factors for negative outcomes of NACD found in this study were multiple NACD procedures, smaller-size calcific deposits, and a longer duration of symptoms prior to NACD. The latter is a well-known negative prognostic factor which is supported by multiple studies [3, 7].

Regarding the effects of multiple procedures on the outcome of NACD doubt could exist about if this variable represents a cause of bad prognosis or simply represents the consequence of attempting the same procedure in non-responding patients. Studies on the effect of multiple procedure on the outcome of NACD are scarce and inconclusive. Farin et al, for example, found that the outcomes of second and third procedures were inferior to the outcome of the primary NACD procedure [16]. On the other hand, studies by del Cura et al and Oudelaar et al found no difference in outcome between shoulders that were treated once and those treated twice or even three times [8, 17]. As the results of this study add evidence to the theory that the number of NACD procedures do negatively affect the outcome of NACD, physicians treating patients with RCCT should be aware of the inferior results of multiple procedures and should manage patient expectations likewise prior to a second NACD procedure.

With regard to the size of calcific deposits, larger-size calcific deposits have been associated with worse outcomes in the conservative treatment of RCCT and after ESWT [3, 18]. In NACD, however, no such associations have been described. This study shows that smaller-size calcific deposits are associated with less improvement of shoulder function. Smaller-size calcific deposits are therefore perhaps less suitable for NACD. A possible explanation for these inferior outcomes is that shoulder complaints in patients with smaller-size calcific deposits are most probable due to more complex inflammatory pathology and not due to a more localized post-inflammatory deposit of one or several “large” calcific deposits. Another explanation for the inferior outcome of NACD in smaller-size calcific deposits could be that patients with smaller-size calcific deposits have less complaints and are therefore less likely to demonstrate improvement. Additional in-depth analyses did however demonstrate that there was no correlation between baseline scores and the size of the calcific deposit (VAS: *r* = − 0.35, *p* = 0.738; SST: *r* = − 0.149, *p* = 0.153; DASH: *r* = 0.099, *p* = 0.345). It seems therefore unlikely that patients with smaller-size calcific deposits have less complaints compared to patients with larger-size calcific deposits which makes it more likely that the shoulder complaints in patients with smaller-size calcific deposits have another cause, such as a more complex inflammatory pathology.

Calcific tendinitis is not always symptomatic as it is found in up to 35% of adults without shoulder complaints [19]. However, larger calcific deposits will sooner or later always result in shoulder complaints according to Bosworth et al [19]. This statement is supported by a study by Louwerens et al in which patients with calcific deposits of > 1.5 cm in length had the highest chance of suffering from symptomatic calcific tendinitis [20]. Based on the findings of this study and the mentioned literature, clinicians should be careful in attributing shoulder complaints to smaller-size calcific deposits and be perhaps more cautious in referring for NACD as the outcomes in patients with smaller-size calcific deposits are less favorable.

Earlier studies on prognostic factors for the effectiveness of NACD, which were all retrospective, found that smoking, female gender, dominant arm involvement, bilateral disease, longer duration of symptoms, and multiple calcifications all were associated with less favorable outcomes [7, 9, 10]. Surprisingly, besides a longer duration of symptoms, none of these factors were associated with improvement of pain, shoulder function, or QoL in this study. These differences can possibly be explained by a difference in outcome measures as a wide range of different outcome measures has been used such as the WORC, DASH, and a binary scale which evaluated whether patients were free of complaints of not. More likely, the differences can be explained by a difference in the duration of follow-up. In a study by de Witte et al, for example, patients were followed up after 14 years [7]. A substantial part of patients reported persisting shoulder complaints, but it is unclear whether the persisting shoulder complaints are due to persisting or recurrent RCCT or due to aging (i.e., degeneration of the rotator cuff) [21].

Twenty-eight possible prognostic factors for the outcome after NACD were evaluated of which only the size of the calcific deposit and the duration of symptoms prior to the intervention were associated with the outcome. Additionally, it is important to acknowledge that NACD is often performed after other therapies have failed. Even more important, little is known on the effect of these previous (failed) therapies on the perceived outcome of NACD. Results of this study demonstrate that previous unsuccessful treatments, in particular ESWT and subacromial injections with corticosteroids, did not affect the outcome of NACD. NACD is therefore also indicated after failure of other therapies for RCCT. Furthermore, other radiological findings such as AC arthrosis and partial thickness rotator cuff tears are often present in RCCT [22]. In the current study, these radiological findings did not affect the outcome of NACD and are therefore not a contra-indication for NACD. Results of the current study also demonstrate that aspiration of the calcific deposit during the NACD procedure had no evident effect on the clinical outcome of NACD. In this study, successful aspiration of calcium could be achieved in 51% of the patients using a 20- or 21-gauge needle. Only two other studies reported on the possibility of aspirating calcium during the NACD procedure. Del Cura et al [17] were able to aspirate calcium in 75% of patients using a 20-gauge needle, whereas Aina et al [23] reported successful aspiration in only 33% of patients using a 22-gauge needle. In literature, a wide range of needle sizes has been reported varying from 18- to 22-gauge [23, 24]. Larger size needles can facilitate the aspiration of calcium but might on the other hand damage the residual tendon fibers [23]. Current literature is inconclusive about the effect of aspiration of calcium during the NACD procedure on the outcome of NACD as the abovementioned studies demonstrate conflicting results [17, 23]. Results of this study demonstrate that successful aspiration of calcium during the NACD procedure is not a prerequisite for a good outcome.

This is the first prospective study evaluating prognostic factors for the effectiveness of NACD. Nevertheless*,* this study has several limitations. First, a relatively large number of patients (39 patients; 26%) was lost to follow-up at 12 months. However, a certain drop-out rate could be expected as others reported drop-out rates of 30% for web-based surveys [25]. Secondly, the study does not have a control group. Although it would have been ideal to have a control group to rule out that the outcome is because of the self-limiting history of RCCT, we believe that, for the purpose of identifying prognostic factors, a large prospective cohort study is of sufficient methodological quality. Finally, the follow-up term of this study was 1 year; this is comparable to other studies [16, 17, 24], but some long-term studies on RCCT show that a substantial part of patients still have persisting shoulder complaints in the long term [7, 26]. More research is needed to gain insight in predictive factors for persisting shoulder complaints in the long term.

In conclusion, NACD provides early pain relief and improvement of shoulder function and quality of life. A good initial response to NACD is associated with a better outcome whereas a longer duration of symptoms prior to NACD, multiple NACD procedures, and smaller-size calcific deposits are associated with inferior outcomes.

## Electronic Supplementary Material

ESM 1 (DOCX 31 kb)

## Abbreviations

DASH: Disabilities of the Arm, Shoulder and Hand questionnaire
ESWT: Extracorporeal shockwave therapy
IRB: Institutional review board
MCID: Minimal clinically important difference
NACD: Needle aspiration of calcific deposits
NSAID: Non-steroidal anti-inflammatory drugs
QoL: Quality of life
RCCT: Rotator cuff calcific tendinitis
SST: Simple shoulder test
VAS: Visual analogue scale
