# Supplementary material for: Prognostic factors for the outcome of needle aspiration of calcific deposits for rotator cuff calcific tendinitis
Source: Eur Radiol. 2020 Mar 5;30(7):4082–90. doi: 10.1007/s00330-020-06669-0 (PMC7305078; doi:10.1007/s00330-020-06669-0)
Supplement: Supplementary file 1 — (DOCX 31 kb) [file 330_2020_6669_MOESM1_ESM.docx]

| **Supplementary file I: univariate analyses for reduction of pain (VAS), improvement of shoulder function (SST and DASH) and improvement of QoL (EQ-5D).** | | | | | | |  | |  | |  | |  | |
| --- | --- | --- | --- | --- | --- | --- | --- | --- | --- | --- | --- | --- | --- | --- |
|  | **Pain** | | | **Function** | | | | | | | **Quality of life** | | | |
|  | **diffVAS** | **p** | **diffSST** | | **p** | **diffDASH** | | **p** | | **diffEQ-5D** | | **p** | |  |
| **Demographic details** |  |  |  | |  |  | |  | |  | |  | |  |
| Gender (male vs. female) | 2,9 (-6,9 to 12,7) | 0,558 | 0,6 (-0,7 to 2,0) | | 0,360 | -2,6 (-9,0 to 3,8) | | 0,426 | | 0,05 (-0,06 to 0,15) | | 0,367 | |  |
| Age | - | 0,793 | - | | 0,443 | - | | 0,361 | | - | | 0,145 | |  |
|  |  |  |  | |  |  | |  | |  | |  | |  |
| **Medical history** |  |  |  | |  |  | |  | |  | |  | |  |
| Duration of symptoms in months | - | 0,441 | - | | 0,143 | - | | 0,495 | | - | | 0,026* | |  |
| Bilateral occurence | -9,5 (-21,8 to 2,8) | 0,128 | 1,1 (-0,7 to 2,8) | | 0,240 | -2,0 (-10,1 to 6,1) | | 0,626 | | -0,03 (-0,16 to 0,10) | | 0,599 | |  |
| Dominant arm affected | -2,6 (-12,6 to 7,4) | 0,602 | -0,5 (-2,0 to 0,9) | | 0,452 | 3,7 (-2,8 to 10,2) | | 0,263 | | -0,05 (-0,15 to 0,06) | | 0,373 | |  |
| Heavy physical work | 3,1 (-6,9 to 13,1) | 0,545 | -0,2 (-1,6 to 1,3) | | 0,826 | 2,9 (-3,6 to 9,4) | | 0,380 | | 0,03 (-0,08 to 0,13) | | 0,615 | |  |
| Absenteeism from work due to shoulder complaints | 3,5 (-9,1 to 16,2) | 0,582 | -1,0 (-2,7 to 0,8) | | 0,271 | 9,3 (-1,1 to 19,7) | | 0,078* | | 0,01 (-0,12 to 0,14) | | 0,909 | |  |
| Smoking | 1,8 (-10,8 to 14,4) | 0,775 | -0,6 (-2,4 to 1,3) | | 0,540 | -0,9 (-9,5 to 7,8) | | 0,846 | | 0,08 (-0,04 to 0,21) | | 0,189 | |  |
| Diabetes | -5,9 (-21,7 to 9,9) | 0,457 | 1,5 (-0,8 to 3,8) | | 0,192 | -8,9 (-19,6 to 1,9) | | 0,104 | | 0,07 (-0,11 to 0,24) | | 0,414 | |  |
|  |  |  |  | |  |  | |  | |  | |  | |  |
| **Previous treatment** |  |  |  | |  |  | |  | |  | |  | |  |
| Analgesics | 4,3 (-5,7 to 14,2) | 0,396 | -0,9 (-2,2 to 0,4) | | 0,173 | 5,6 (-0,4 to 11,6) | | 0,067* | | 0,06 (-0,05 to 0,16) | | 0,282 | |  |
| Physiotherapy | 8,1 (-3,1 to 19,3) | 0,156 | -0,8 (-2,4 to 0,8) | | 0,325 | 8,6 (1,3 to 15,8) | | 0,021* | | -0,01 (-0,13 to 0,11) | | 0,837 | |  |
| Shockwave therapy | 4,8 (-9,1 to 18,7) | 0,495 | 0,3 (-1,7 to 2,3) | | 0,743 | 1,7 (-7,4 to 10,9) | | 0,711 | | -0,09 (-0,24 to 0,06) | | 0,215 | |  |
| Subacromial injection | 3,6 (-6,1 to 13,3) | 0,465 | 1,1 (-0,3 to 2,4) | | 0,132 | -5,3 (-11,5 to 1,0) | | 0,100 | | -0,05 (-0,16 to 0,05) | | 0,310 | |  |
|  |  |  |  | |  |  | |  | |  | |  | |  |
| **Sonographic findings** |  |  |  | |  |  | |  | |  | |  | |  |
| Subacromial bursitis | -3,7 (-16,5 to 9,0) | 0,562 | -0,5 (-2,3 to 1,4) | | 0,626 | -2,3 (-8,6 to 4,0) | | 0,466 | | -0,08 (-0,22 to 0,07) | | 0,291 | |  |
| Partial thickness rotator cuff tear | 7,0 (-3,9 to 17,9) | 0,205 | 0,2 (-1,4 to 1,8) | | 0,795 | 1,3 (-6,1 to 8,6) | | 0,735 | | -0,01 (-0,13 to 0,11) | | 0,848 | |  |
| Subacromial impingement | 14,2 (-4,2 to 32,7) | 0,128 | -1,8 (-4,3 to 0,7) | | 0,144 | 11,1 (-0.9 to 23,1) | | 0,068* | | -0,08 (-0,28 to 0,12) | | 0,446 | |  |
|  |  |  |  | |  |  | |  | |  | |  | |  |
| **Radiographic findings** |  |  |  | |  |  | |  | |  | |  | |  |
| Size in mm | - | 0,273 | - | | 0,029* | - | | 0,031* | | - | | 0,018* | |  |
| Numer of calcific deposits (one vs. multiple) | -3,5 (-13,7 to 6,6) | 0,492 | 0,5 (-1,0 to 2,0) | | 0,492 | 0,0 (-6,7 to 6,7) | | 0,991 | | 0,01 (-0,11 to 0,12) | | 0,904 | |  |
| Gartner & Heyer classification |  |  |  | |  |  | |  | |  | |  | |  |
| *Type I* | 29 | 0,375 | -3,5 | | 0,649 | 18 | | 0,472 | | 0,08 | |  | |  |
| *Type II* | 37 |  | -4,1 | |  | 19 | |  |  | 0,16 | | 0,391 | |  |
| *Type III* | 30 |  | -3,3 | |  | 13 | |  |  | 0,08 | |  | |  |
| Location according to Ogon et al. In mm | - | 0,982 | - | | 0,651 | - | | 0,591 | | - | | 0,517 | |  |
| Features of AC osteoarthrosis | -6,3 (-16,1 to 3,6) | 0,207 | 0,3 (-1,2 to 1,7) | | 0,733 | -2,8 (-9,3 to 3,7) | | 0,390 | | -0,04 (-0,14 to 0,07) | | 0,466 | |  |
|  |  |  |  | |  |  | |  | |  | |  | |  |
| **Findings during NACD** |  |  |  | |  |  | |  | |  | |  | |  |
| Aspiration of calficic deposit during procedure | -0,2 (-10,1 to 9,8) | 0,975 | 0,5 (-0,9 to 1,9) | | 0,475 | -1,7 (-8,2 to 4,8) | | 0,614 | | 0,07 (-0,04 to 0,17) | | 0,196 | |  |
|  |  |  |  | |  |  | |  | |  | |  | |  |
| **Post NACD** |  |  |  | |  |  | |  | |  | |  | |  |
| Analgesics during first 6 weeks post NACD | 5,9 (-4,4 to 16,3) | 0,259 | -0,9 (-2,3 to 0,7) | | 0,250 | 4,4 (-2,3 to 11,1) | | 0,195 | | -0,08 (-0,20 to 0,03) | | 0,141 | |  |
| Physiotherapy during first 6 weeks post NACD | 7,2 (-3,8 to 18,2) | 0,199 | -1,0 (-2,6 to 0,6) | | 0,208 | 4,8 (-2,3 to 12,0) | | 0,183 | | 0,07 (-0,06 to 0,18) | | 0,320 | |  |
|  |  |  |  | |  |  | |  | |  | |  | |  |
| **Number of NACD procedures** |  |  |  | |  |  | |  | |  | |  | |  |
| *One vs. multiple procedures* | 15,5 (5,1 to 25,9)) | <0,01* | 1,8 (0,3 to 3,3) | | 0,023* | 8,2 (1,4 to 15,1) | | 0,019* | | 0,10 (-0,01 to 0,21) | | 0,088* | |  |
|  |  |  |  | |  |  | |  | |  | |  | |  |
| **Results 3 months postNACD** |  |  |  | |  |  | |  | |  | |  | |  |
| DiffVAS | - | <0,01* | - | | 0,265 | - | | 0,316 | | - | | 0,150 | |  |
| DiffSST | - | 0,118 | - | | <0,01* | - | | <0,01* | | - | | 0,005* | |  |
| DiffDASH | - | <0,01* | - | | <0,01* | - | | <0,01* | | - | | 0,063* | |  |
| Average differences are presented with the 95% confidence interval within the parentheses. -: no average difference could be presented as correlation coefficients were calculated. *: p<0.10 and hence inclusion in multivariate analysis. | | | | | | | | | | | | | | |
